# Supplementary material for: Electronic stripe patterns near the fermi level of tetragonal Fe(Se,S)
Source: NPJ Quantum Mater. 2023 Oct 19;8(1):60. doi: 10.1038/s41535-023-00592-5 (PMC11041788; doi:10.1038/s41535-023-00592-5)
Supplement: Supplementary file 1 — Supplementary Information [file 41535_2023_592_MOESM1_ESM.pdf]

Supplementary Information for Electronic Stripe Patterns Near the Fermi Level of Tetragonal Fe(Se,S)

M. Walker,[1,2,3] K. Scott,[2,3] T. J. Boyle,[1,2,3] J. K. Byland,[1] S. Bötzel,[4] Z. Zhao,[1] R. P. Day,[5,6] S. Zhdanovich,[5,6] S. Gorovikov,[7] T. M. Pedersen,[7] P. Klavins,[1] A. Damascelli,[5,6] I. M. Eremin,[4] A. Gozar,[2,3] V. Taufour,[1] and E. H. da Silva Neto[1,2,3,8,*]

[1]*Department of Physics and Astronomy, University of California, Davis, California 95616, USA*

[2]*Department of Physics, Yale University, New Haven, Connecticut 06520, USA*

[3]*Energy Sciences Institute, Yale University, West Haven, Connecticut 06516, USA*

[4]*Institut für Theoretische Physik III, Ruhr-Universität Bochum, D-44801 Bochum, Germany*

[5]*Quantum Matter Institute, University of British Columbia, Vancouver, BC V6T 1Z4, Canada*

[6]*Department of Physics & Astronomy, University of British Columbia, Vancouver, BC V6T 1Z1, Canada*

[7]*Canadian Light Source, Saskatoon, Saskatchewan S7N 2V3, Canada*

[8]*Department of Applied Physics, Yale University, New Haven, Connecticut 06520, USA*

* Corresponding Author: eduardo.dasilvaneto@yale.edu

Supplementary Methods

Supplementary Table 1. Parameters of STM measurements on $FeSe_{1-x}S_x$ shown in the main text.

Figure	x	Bias (mV)	Current (pA)	Scan size (nm^2)	Grid size	Modulation amplitude (mV)
1c	0	-20	500	10x10	256x256	N/A
1e	0.19	-10	1500	80x80	1024x1024	N/A
1b (green) 1d	0	-42	200	170x170	256x256	1
1b (inset)	0	-20	200	20x20	32x32	0.25
1b (purple) 1f 5a 5d-i 6	0.19	-50	450	170x170	256x256	0.75
2\{dhl} 3 4e-h 5bc	0.19	-50	450	46x46	256x256	0.75
2dhl 4e-h	0.19	-60	450	46x46	256x256	0.75
S4	0.23	-42	250	170x170	256x256	1.5

ARPES measurements were carried out on the Quantum Materials Spectroscopy Centre beamline at the Canadian Light Source, with vertically and horizontally polarized light with photon energy ranging from 18 to 40 eV (see figure captions). Samples were cleaved *in situ* and measured at pressure lower than 5×10^{-11} Torr and a temperature of 9 K. The combined beamline-analyzer (Scienta R4000) resolutions in angle and energy are better than 0.1° and 9 meV, respectively, for the combinations of beamline parameters (monochromator grating, exit slit, temperature, photon energy) used.

We briefly present our theoretical model used to calculate QPI patterns of Fig. 4a-d in the main text. In the normal state of $FeSe_{0.77}S_{0.23}$, the electronic Hamiltonian near the Γ-$(0,0,0)$ and Z-point $(0,0,\pi)$ reads [1, 2]

$$H_{\Gamma/\mathrm{Z}} = \sum_{\mathbf{k}} \Psi^{\dagger}_{\Gamma/\mathrm{Z}}(\mathbf{k}) \left[h^{0}_{\Gamma/\mathrm{Z}}(\mathbf{k}) + h^{\mathrm{SOC}}_{\Gamma/\mathrm{Z}}(\mathbf{k}) \right] \Psi_{\Gamma/\mathrm{Z}}(\mathbf{k}), \tag{1}$$

where $\Psi_{\Gamma/\mathrm{Z}} = (\mathrm{d}_{\mathrm{yz},\uparrow}, \mathrm{d}_{\mathrm{xz},\uparrow}, \mathrm{d}_{\mathrm{yz},\downarrow}, \mathrm{d}_{\mathrm{xz},\downarrow})^{\mathrm{T}}$ is the four component spinor with the momentum label ($\mathbf{k}$) being implicit and measured as deviation from the Γ- or Z-point. The term

$$h^{0}_{\Gamma/\mathrm{Z}}(\mathbf{k}) = \begin{pmatrix} \epsilon_{\mathrm{h}} - \frac{k^2}{2m} - \frac{b}{2}(k_{\mathrm{x}}^2 - k_{\mathrm{y}}^2) & -2ck_{\mathrm{x}}k_{\mathrm{y}} \\ -2ck_{\mathrm{x}}k_{\mathrm{y}} & \epsilon_{\mathrm{h}} - \frac{k^2}{2m} + \frac{b}{2}(k_{\mathrm{x}}^2 - k_{\mathrm{y}}^2) \end{pmatrix} \otimes \sigma_0 \tag{2}$$

models the electronic dispersion while the effect of spin-orbit coupling is captured by

$$h^{\mathrm{SOC}}_{\Gamma/Z}(\mathbf{k}) = \frac{\lambda_{\mathrm{SOC}}}{2}\tau_2 \otimes \sigma_3. \tag{3}$$

Here Pauli matrices σ_{i} and τ_{i} act on the spin and orbital space, respectively. The fitting parameters for the electronic states near the Γ and Z points, which enter into Eq. 2, are listed in Table 2. The resulting band dispersion in direct comparison with our ARPES data is shown in Supplementary Fig. 1a,b for the Γ-point and in Supplementary Fig. 1c,d for the Z-point.

For the electron pockets near the X and Y points of the one-iron Brillouin zone which are folded to the A-point in the two-iron unit cell, the Hamiltonian has the form [1, 2]

$$H_{\mathrm{A}} = \sum_{\mathbf{k}} \left(\Psi^{\dagger}_{\mathrm{Y}}(\mathbf{k}), \Psi^{\dagger}_{\mathrm{X}}(\mathbf{k})\right) \begin{pmatrix} h_Y(\mathbf{k}) & 0 \\ 0 & h_X(\mathbf{k}) \end{pmatrix} \begin{pmatrix} \Psi_Y(\mathbf{k}) \\ \Psi_X(\mathbf{k}) \end{pmatrix}. \tag{4}$$

The spinors are $\Psi_{\mathrm{Y}} = \left(\mathrm{d}_{\mathrm{xz},\uparrow}, \mathrm{d}^{\mathrm{Y}}_{\mathrm{xy},\uparrow}, \mathrm{d}_{\mathrm{xz},\downarrow}, \mathrm{d}^{\mathrm{Y}}_{\mathrm{xy},\downarrow}\right)^{\mathrm{T}}$ and $\Psi_{\mathrm{X}} = \left(\mathrm{d}_{\mathrm{yz},\uparrow}, \mathrm{d}^{\mathrm{X}}_{\mathrm{xy},\uparrow}, \mathrm{d}_{\mathrm{yz},\downarrow}, \mathrm{d}^{\mathrm{X}}_{\mathrm{xy},\downarrow}\right)^{\mathrm{T}}$, where again the ($\mathbf{k}$) dependence is implicit and considered as deviation from the (π, π, π) A-point. Here the electronic dispersion reads

$$H_{\mathrm{X,Y}} = \begin{pmatrix} \epsilon_1 + \frac{k^2}{2m_1} \mp \frac{a_1}{2}(k_{\mathrm{x}}^2 - k_{\mathrm{y}}^2) & -\mathrm{i}v_{\mathrm{X,Y}}(\mathbf{k}) \\ \mathrm{i}v_{\mathrm{X,Y}}(\mathbf{k}) & \epsilon_3 + \frac{k^2}{2m_3} \mp \frac{a_3}{2}(k_{\mathrm{x}}^2 - k_{\mathrm{y}}^2) \end{pmatrix} \otimes \sigma_0, \tag{5}$$

where $v_{\mathrm{X}}(\mathbf{k}) = \sqrt{2}vk_{\mathrm{y}} + \frac{p_1}{\sqrt{2}}\left({k_{\mathrm{y}}}^3 + 3k_{\mathrm{y}}{k_{\mathrm{x}}}^2\right) - \frac{p_2}{\sqrt{2}}k_{\mathrm{y}}\left({k_{\mathrm{x}}}^2 - {k_{\mathrm{y}}}^2\right)$ and $v_{\mathrm{Y}}(\mathbf{k}) = v_{\mathrm{X}}(k_{\mathrm{y}}, k_{\mathrm{x}})$. At this point we neglect the effect of spin-orbit coupling at the A-point, which would lead to hybridization of X and Y pockets, but is found to be weaker than near the center of the zone [3]. The same is true for the less pronounced three-dimensionality of the Fermi surface pockets at the zone corners[4, 5], so for simplicity we keep the parametzization of the A-point throughout. The fitting parameters for the A-point electronic states that enter into Eq. 5 are listed in Tab. 2. The resulting band dispersion in comparison with our ARPES data is shown in Supplementary Fig. 1e,f.

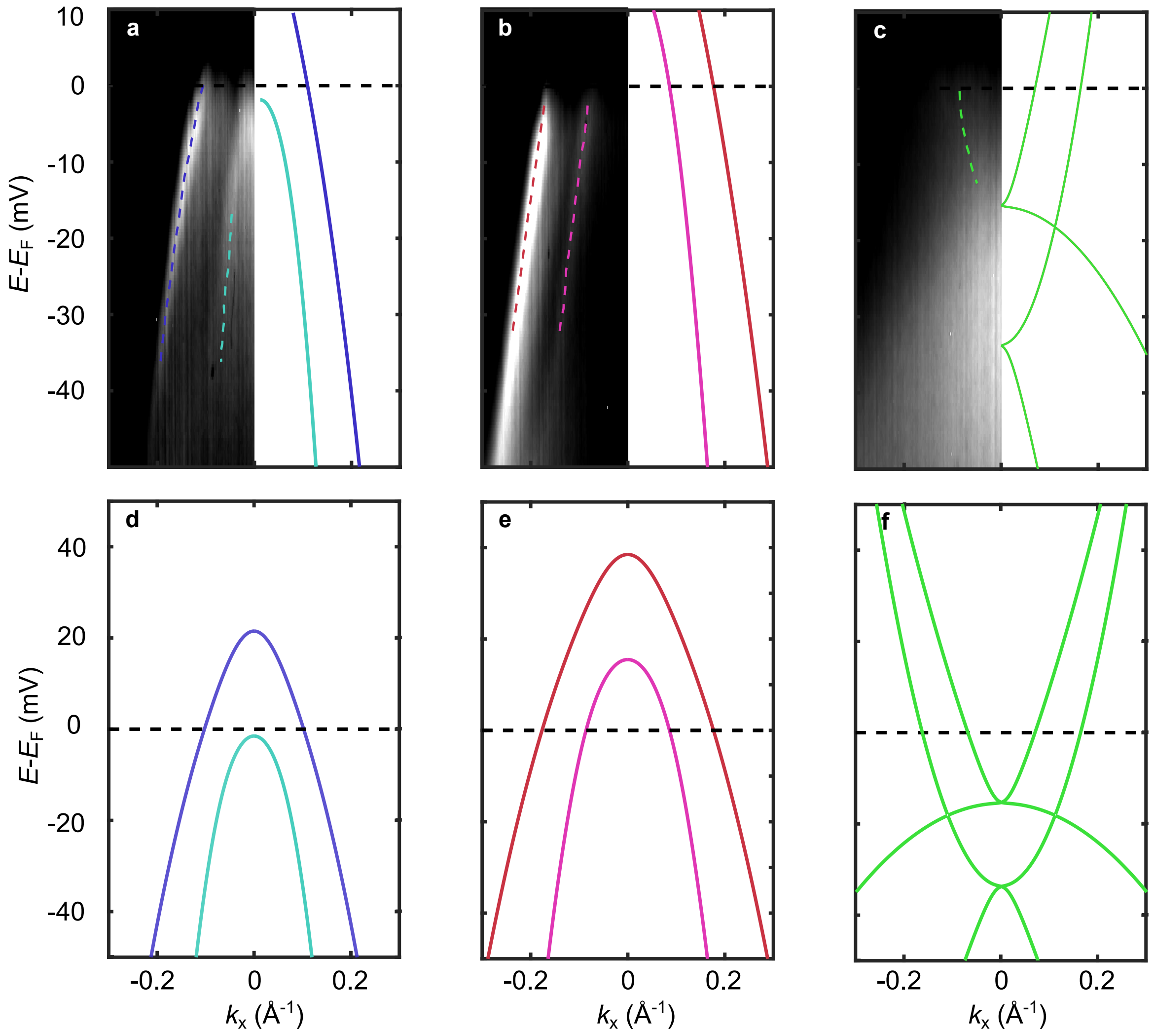

Supplementary Figure 1. **a-c** Left side: ARPES image near the Γ, Z, A points of $FeSe_{0.77}S_{0.23}$. The lines on the left are determined by fitting the momentum distribution curves (MDCs) to Lorentzian peaks. Right side: modeled band structure adjusted to best match the bands measured by ARPES. **d-f** Same calculations shown over the full k_x range and a broader energy range. ARPES measurements at the Γ, Z, and A were taken with photon energies $PE = 38\,\mathrm{eV}$, $PE = 19\,\mathrm{eV}$, and $PE = 18\,\mathrm{eV}$, respectively.

Supplementary Table 2. Fitting parameters for the states at the high-symmetry points fitted to match ARPES and QPI data of Supplementary Fig. 2(a-d) and Fig. 4, respectively.

	Γ	Z	
ϵ_{h}	10	27	meV
$\frac{1}{2m_{\mathrm{h}}}$	2661	1848	meVÅ^2
b	2624	1822	meVÅ^2
c	-1312	-911	meVÅ^2
$\lambda_{\mathrm{SOC}}^{\mathrm{h}}$	15	15	meV

	A	
ϵ_1	-3.6	meV
ϵ_3	-25.6	meV
$\frac{1}{2m_1}$	4.6	meVÅ^2
$\frac{1}{2m_3}$	603	meVÅ^2
α_1	441	meVÅ^2
α_3	-1308.4	meVÅ^2
v	-221.2	meVÅ
p_{z_1}	-800.3	meVÅ^3
p_{z_2}	-68.1	meVÅ^3

We calculate the QPI energy-momentum structure of the local density of states in the Born limit given by the convolution of the bare Green's functions dressed by the scattering matrix of a nonmagnetic impurity, $\hat{V}$.

$$\rho(\omega, \mathbf{q}) = -\frac{1}{\pi}\mathrm{ImTr}\sum_{\mathbf{k}}\hat{G}_{\mathbf{k}}(\omega) - \frac{1}{\pi}\mathrm{ImTr}\sum_{\mathbf{k}}\hat{G}_{\mathbf{k}}(\omega)\hat{V}\hat{G}_{\mathbf{k+q}}(\omega) \tag{6}$$

Here the corresponding bare Green's function in momentum space is given by $\hat{G}_{\mathbf{k}}(\omega) = [(\omega + \mathrm{i}\delta) - h(\mathbf{k})]^{-1}$, where

$$h(\mathbf{k}) = \begin{pmatrix} h^0_{\Gamma/\mathrm{Z}}(\mathbf{k}) + h^{\mathrm{SOC}}_{\Gamma/\mathrm{Z}} & 0 & 0 \\ 0 & h_{\mathrm{Y}}(\mathbf{k}) & 0 \\ 0 & 0 & h_{\mathrm{X}}(\mathbf{k}) \end{pmatrix}, \tag{7}$$

and $\hat{V} = \mathbb{I}_{3\times3} \otimes (V_0\tau_0) \otimes \sigma_0$ refers to the intraorbital non-magnetic scattering potential leading to small $\mathbf{q}$ intra- and interband scattering processes between hole and electron states, respectively. We use Eq. 6 to calculate the resulting QPI patterns as a function of bias voltage (ω) and momentum ($\mathbf{q}$) shown in Fig. 4a-d of the main text for the $k_{\mathrm{z}} = 0$ and $k_{\mathrm{z}} = \pi$ cuts.

Supplementary Figures

dI/dV of $FeSe_{1-x}S_x$ samples over multiple energies. dI/dV measurements displayed in the main text show the presence of dispersive and non-dispersive spatial modulations. For completeness, we show dI/dV maps of FeSe and $FeSe_{0.81}S_{0.19}$ over additional energies in Supplementary Fig. 2 and 3, respectively. Additionally, we show the Fourier transforms without any symmetrization and with two-fold ($x = 0$) or four-fold ($x = 0.19$) symmetrization side-by-side. Based on the four-fold symmetry present in Supplementary Fig. 3i-l, all Fourier transforms on $FeSe_{0.81}S_{0.19}$ shown in the main text are four-fold symmetrized to improve signal to noise.

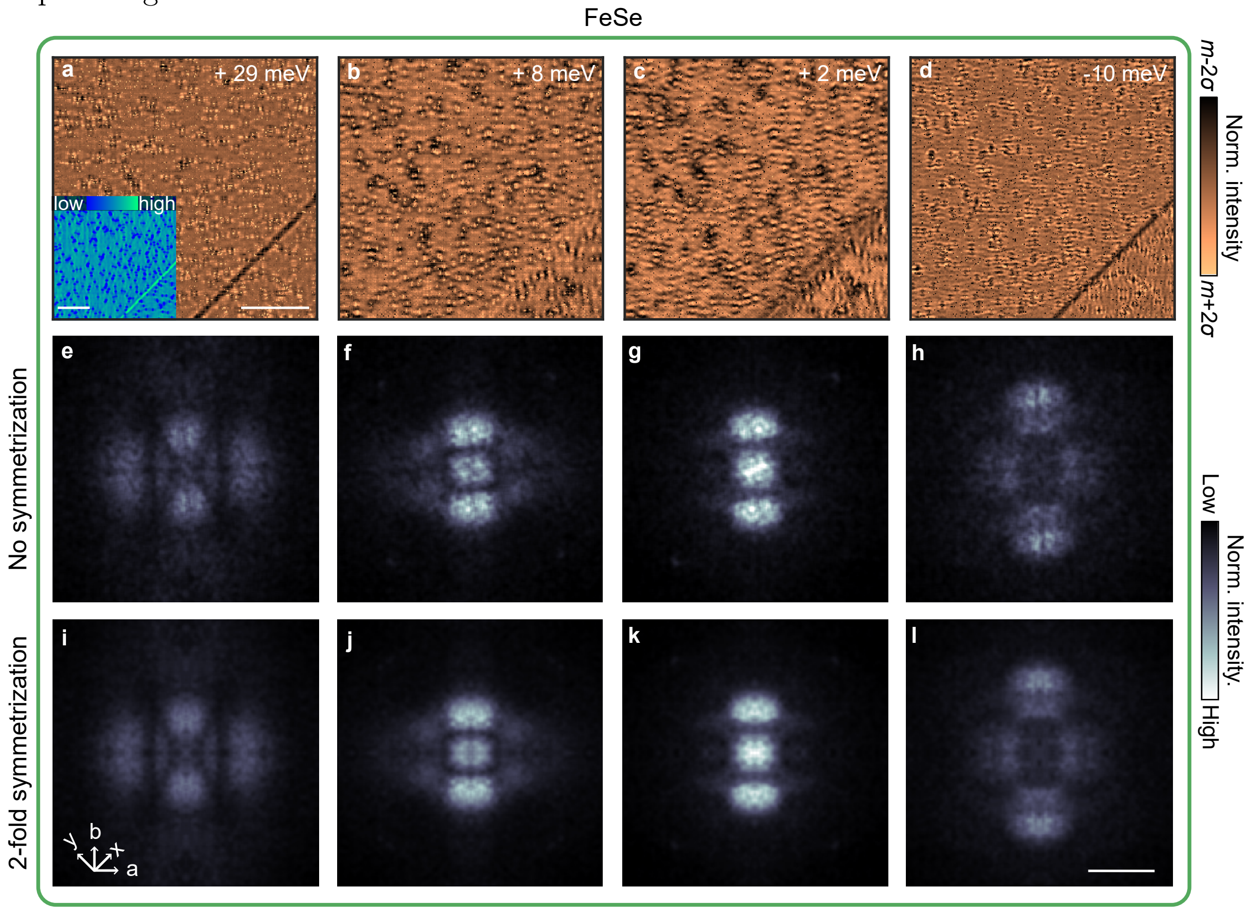

Supplementary Figure 2. **dI/dV maps of FeSe at additional energies.** **a-d** dI/dV maps on FeSe, at selected energies. The inset in **a** is the topography that was recorded simultaneously with the dI/dV in **a-d**; both scale bars represent 50 nm. The dI/dV maps were normalized by their standard deviation after subtracting the mean. **e-h** Fourier transform of the real-space images in **a-d** with no symmetrization. **i-l** Same as **e-h** but with two-fold symmetrization. Scale bar is 0.2 Å^{-1}.

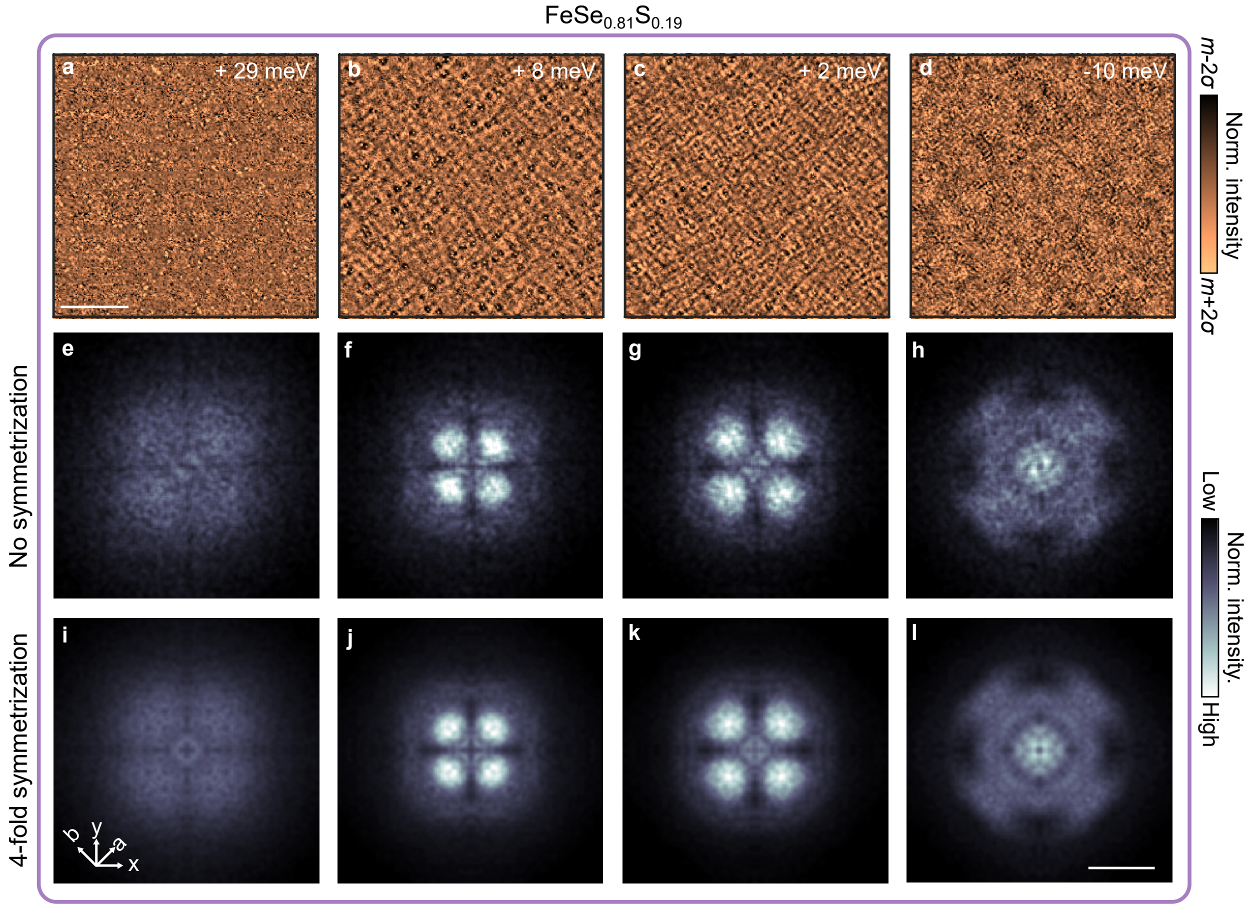

Supplementary Figure 3. **dI/dV maps of FeSe$_{0.81}$S$_{0.19}$ at additional energies. a-d** dI/dV maps on FeSe$_{0.81}$S$_{0.19}$, at selected energies. Scale bar is 50 nm. The maps were normalized by their standard deviation after subtracting the mean. **e-h** Fourier transform of the real-space images in **a-d** with no symmetrization. **i-l** Same as **e-h** but with four-fold symmetrization. Scale bar is $0.2\,\text{Å}^{-1}$.

Measurements over additional Sulfur concentrations. In the main text we describe STS measurements that reveal the presence of electronic stripes in $FeSe_{0.81}S_{0.19}$. We found that the STS signatures of these stripes also appear in $FeSe_{0.77}S_{0.23}$, see Supplementary Figure 4.

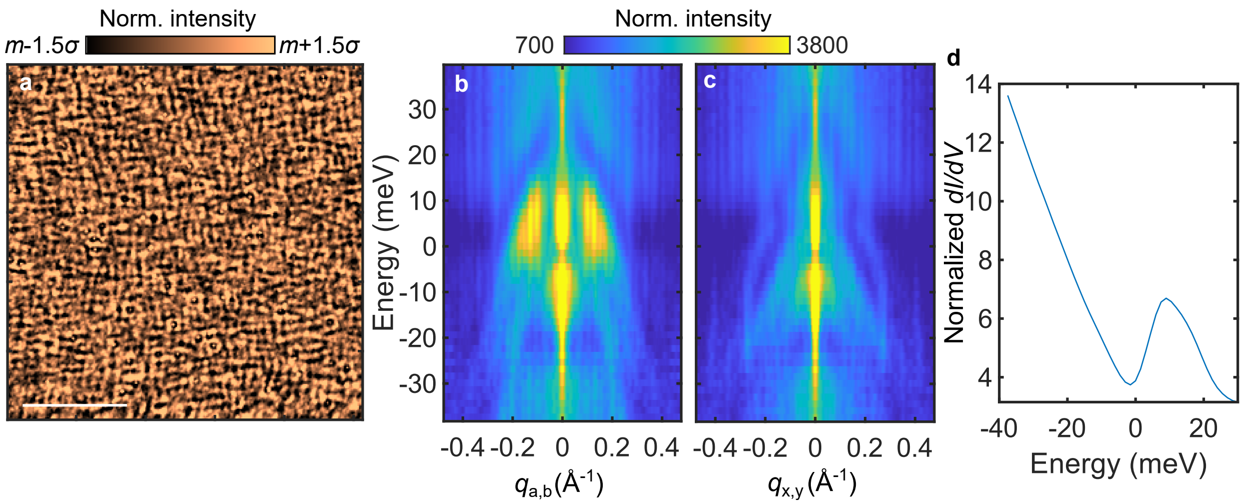

Supplementary Figure 4. **Measurements of additional sulfur concentrations.** **a** Real-space dI/dV map at 6 meV of a $FeSe_{0.77}S_{0.23}$ sample taken with similar measurement conditions to those in row 3 of Table 1. The scale bar is 50 nm. **b,c** Energy-momentum structure of the modulations along the smallest Fe-Fe direction, $q_{a,b}$ (**b**), and the smallest Se-Se direction, $q_{a,b}$ (**c**) obtained from the FTs of the measured dI/dV maps. **d** Representative spatially averaged differential conductance (dI/dV) spectra.

Supplementary Notes

Fits to the two features in Figure 5i. Linecuts taken at the selected energies for the two regions were fitted with Gaussian peaks plus a small constant offset (Supplementary Figure 5). The central area ($q \approx 0$) was fitted by one or two Gaussian peaks. To characterize the QPI features at finite q values we used two symmetric peaks for the left panel and two additional peaks for the right panel to include the small intensity feature around $q \approx 0.23$.

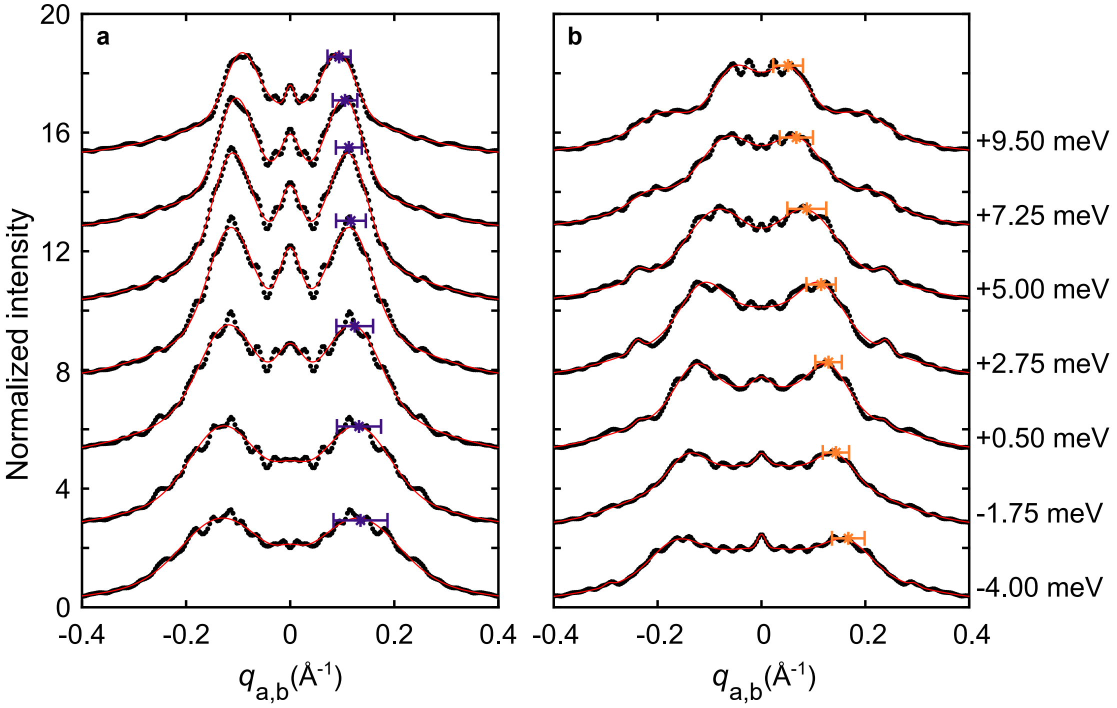

Supplementary Figure 5. **Fits to the two features in Figure 5i. a**,**b** Constant energy linecuts (black dots) from Fig. 5i of the main text at the energies indicated on the right of **b**. Red lines are fits of the data, blue and orange stars indicate the peak position extracted from the fit with estimated error. Error bars come from the HWHM of the fitted peak.

Image filtering. The data in Fig. 6b of the main text shows a dI/dV image of the stripe modulations after filtering. Supplementary Figure 6 compares the filtered and unfiltered images of the entire scan area. The filter is applied in Fourier space and it is composed of four Gaussian peaks centered around the stripe peaks in Fourier transform of the original image.

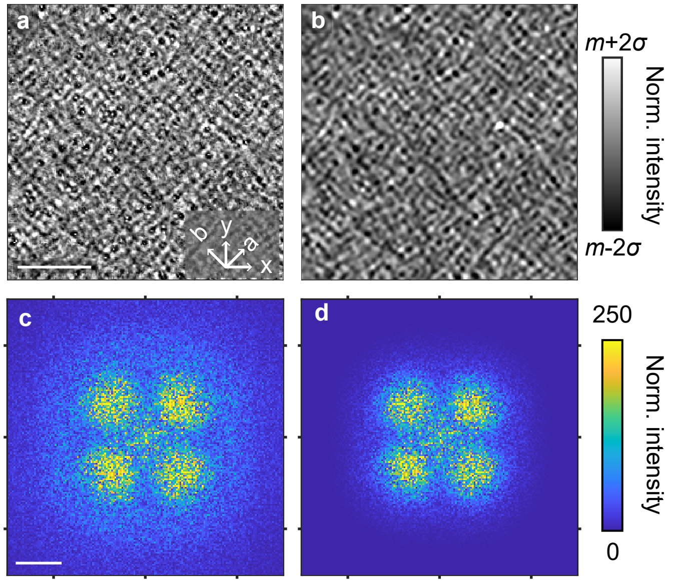

Supplementary Figure 6. **Image Filering.** **a**,**b** Raw and filtered dI/dV map on $FeSe_{0.81}S_{0.19}$ at 4 mV, normalized by the mean of the image. Scale bar is 50 nm. **c**,**d** Fourier transform of the images in **a** and **b** respectively. The filtered image is obtained by applying a Gaussian filter centered at the locations of the four peaks that compose the 0.12 Å^{-1} modulations, as can be seen by comparing **c** and **d**. Scale bar is 0.1 Å^{-1}

Correlation between FeVs locations and domain boundaries. The location of FeVs was determined at an energy (+29 meV) where QPI is weak and the FeVs stand out as bright spots in the dI/dV maps (see Supplementary Fig. 7a). We identified any signal larger than 1.5 standard deviations above the mean value of the dI/dV as an FeV. Positions of FeVs were given a value of one, and zero was assigned elsewhere.

To create the domain map shown in Fig. 6c,d of the main text, the dI/dV image is multiplied by a modulation with wavelength equal to the wavelength of the $q = 0.12\,\text{Å}^{-1}$ electronic stripes in the q_a and q_b directions separately. This product is Fourier transformed, multiplied by a Gaussian window and then inverse Fourier transformed back to real space. In this way we obtain two spatial maps corresponding to the intensities of the these modulations along q_a and q_b. The intensity maps for the two directions are subtracted to get the red/blue domain map in Fig. 6c of the main text, where positive (negative) values indicate modulations primarily along q_b (q_a) are blue (red). The domain boundaries have a near zero value in this map, so to get the domain map shown in Fig. 6d of the main text, the absolute value is taken, divided by the maximum value, and subtracted from unity. Now, unity indicates the position of domain boundaries.

We define a correlation strength between the locations of FeVs and domain boundaries via the product of their corresponding real-space maps. This correlation strength is then compared to the control parameter obtained by randomly placing impurities similar in size and quantity to what is found in our experiment. Doing this for 1000 trials of randomly located impurities, we find a distribution of correlation strengths (Supplementary Fig. 7c). We also find that the correlation strength from the experimentally determined FeVs locations is just over two standard deviations above the mean of this distribution.

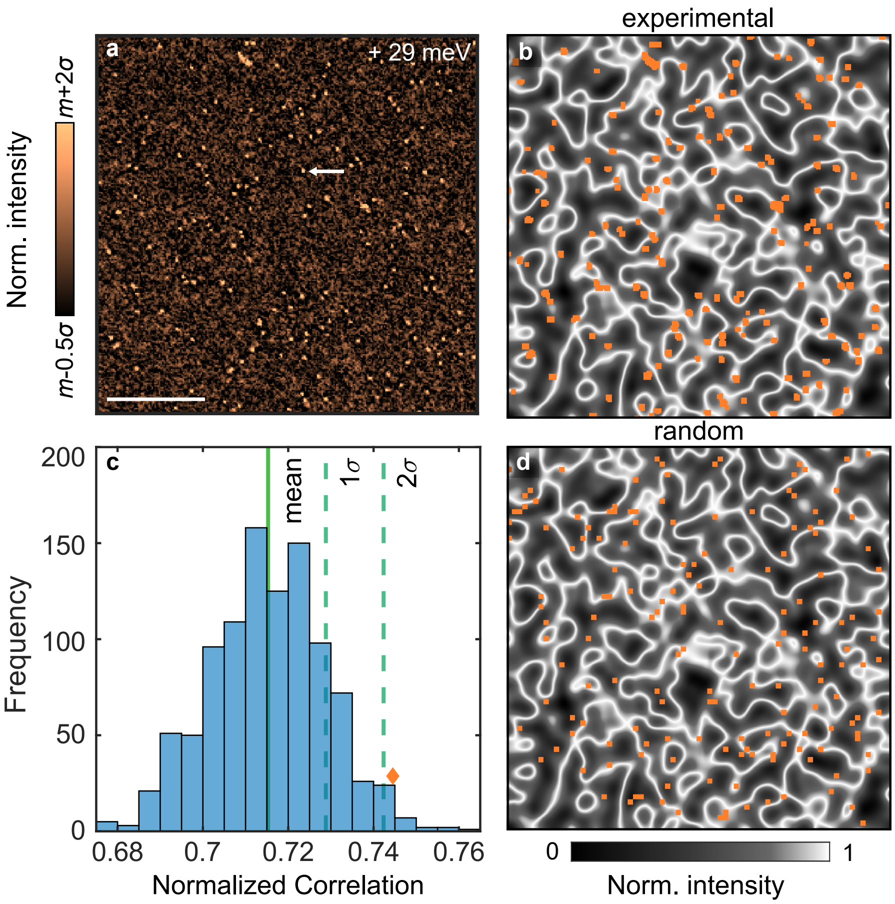

Supplementary Figure 7. **Correlation between FeVs locations and domain boundaries.** **a** dI/dV map of $FeSe_{0.81}S_{0.19}$ at +29 meV. The scale bar represents 50 nm and the white arrow indicates one FeV. The low end of color scale is saturated to highlight the FeVs. **b** Map showing the domain boundaries (white lines) from Fig. 6c of the main text with locations of FeVs overlaid (orange). The FeV positions are determined experimentally at +29 meV. **c** Histogram of the correlation between the location of FeVs and the domain boundaries in d, obtained from 1000 simulated sets of 150 randomly located FeVs. The orange diamond indicates the correlation between the experimental iron-vacancy locations and the domain boundaries. The solid green line represents the mean of the distribution and the two dashed lines represent 1 and 2 standard deviations, σ. **d** Similar to **b** but the orange squares represent a random distribution of FeVs whose correlation with domain boundaries is near the mean correlation.

[1] V. Cvetkovic & O. Vafek. Space group symmetry, spin-orbit coupling, and the low-energy effective Hamiltonian for iron-based superconductors. *Phys. Rev. B* **88**, 13 (2013).

[2] L. C. Rhodes, J. Böker, M. A. Müller, M. Eschrig & I. M. Eremin. Non-local d_{xy} nematicity and the missing electron pocket in FeSe. *npj Quantum Mater.* **6**, 45 (2021).

[3] Y. Zhang *et al.* Superconducting Gap Anisotropy in Monolayer FeSe Thin Film. *Phys. Rev. Lett.* **117**, 11 (2016).

[4] M. D. Watson *et al.* Evidence for unidirectional nematic bond ordering in FeSe. *Phys. Rev. B* **94**, 20 (2016).

[5] Y. S. Kushnirenko *et al.* Three-dimensional superconducting gap in FeSe from angle-resolved photoemission spectroscopy. *Phys. Rev. B* **97**, 18 (2018).
